# The Stockholm Syndrome of Cancer: Fibroblasts as a Powerful Shield against Colorectal Cancer Therapy

**DOI:** 10.3390/cancers15020491

**Published:** 2023-01-13

**Authors:** Samuele Tardito, Maria Raffaella Zocchi, Roberto Benelli

**Affiliations:** 1 SSD Oncologia Molecolare e Angiogenesi, IRCCS Ospedale Policlinico San Martino, 16132 Genova, Italy; 2 Divisione Di Immunologia, Trapianti e Malattie Infettive, IRCCS Istituto Scientifico San Raffaele, 20132 Milano, Italy

## 1. Introduction

Fibroblasts are incredible cells. Scattered throughout the body, filling any gap among organs and tissues, they were initially imagined as a monotonous and passive cell population that, at best, could produce the extracellular matrix necessary to cement the body components. Year after year, scientific research has accumulated a huge amount of evidence that points to fibroblasts as masters of physiological and pathological processes, while the definition of “fibroblast” has become more and more elusive, due to the lack of specific markers and the variety of different subpopulations.

When trying to identify the original vocation of fibroblasts, we can affirm that they are the guardians of tissue homeostasis [1,2]. Along with leucocytes and endothelial cells, which share the same embryonal origin, they immediately react to any local imbalance to restore equilibrium. Fibroblasts are also actively involved in maintaining the steady-state level of several organs. For example, in the gut, they contribute to stem-cell niche subsistence and to promote the subsequent steps of epithelial differentiation [3].

During a famous bank robbery in Stockholm, the hostages developed positive attitudes towards their jailer, spontaneously protecting him with their bodies when he finally surrendered to police. This paradoxical reaction was named Stockholm syndrome, and fits the behavior of cancer-associated fibroblasts (CAFs) well. In 1986, Dvorak correctly defined cancer as “a wound that does not heal” [4], and fibroblasts support tumor cells accordingly. Indeed, many epithelial tumors, at least in their benign stage, are histologically predisposed to be protected and enveloped by fibroblasts. With the exception of very small molecules, nothing can reach the epithelium from the bodily fluids if not filtered by its underlying fibroblasts. While cancer causes local tissue damage, invading and breaking tissue continuity, CAFs support regeneration by providing a cocktail of mediators that can improve tumor growth, angiogenesis, immune escape and invasion itself [5,6]. This phenomenon is particularly evident in the “stroma-rich” form of colorectal cancer (CRC), where the population of CAFs largely outnumbers cancer cells and can actively mediate their metastatic dispersion, causing a poor prognosis [7].

A particularly upsetting feature of CAFs is their ability to protect cancer cells from therapy. This phenomenon is particularly relevant, but infrequently investigated.

While most mechanisms mediating CAFs protection of tumor cells have been described as acting locally, the systemic reaction of fibroblast populations to chemotherapy is rarely considered. An interesting exception is the study by Loupakis et al. [8], which monitored the plasma levels of epithelial growth factor receptor (EGFR) ligands during cetuximab plus irinotecan therapy in 45 irinotecan-refractory metastatic CRC (mCRC) patients. In particular, epithelial growth factor (EGF) and Amphiregulin (AREG) levels were quantified by ELISA before and 1 h after therapy infusion. The same measurements were performed in the second session of therapy, 15 days later. KRAS^12,13,61^ mutations (11 patients) were assessed for the differential analysis of data.

The response of patients with wild-type KRAS mCRC was unique and particularly interesting: AREG incremented soon after therapy and never returned to the baseline levels in the following time-points. EGF showed intermittent spikes, with drops soon after therapy and peaks in the breaks between administrations. Overall survival and progression-free survival were negatively linked to an early (1 h) increase of AREG or a 15 d increase of EGF. The involvement of increased EGFR ligands could partly explain the resistance to Cetuximab therapy of some KRAS wild-type mCRC, while the source of these growth factors was not investigated. AREG is a well-known autocrine growth factor produced by tumor cells in CRC, while EGF is not. Moreover, the completely different kinetics of AREG and EGF after therapy indicates independent sources.

## 2. CAF Counterbalances Cetuximab, Increasing EGF Levels

A possible answer to this puzzle comes from the interesting study by C.M. Garvey et al. [9], which points to CAFs as major mediators. The authors showed that CRC CAFs express EGFR both in the original tumor lesion and, once explanted, in vitro. Accordingly, Cetuximab-based therapy could attack both tumor cells and CAFs. However, in vitro, Cetuximab did not alter CAF viability in contrast to CRC cell lines. This observation suggests that a first shielding exerted by CAFs could be the seizure of a quote of free Cetuximab, reducing its overall active concentration in the tumor stroma.

By in vitro testing of the cytostatic and cytotoxic effects of Cetuximab in the presence of various CAF:CRC cell ratios, the authors showed that a 30% CAF population was enough to prevent CRC epithelial cell crisis, with increased shielding effects at higher ratios. CAFs exerted a double effect, both preserving epithelial CRC cell proliferation and reducing cell death. The conditioned media of CAFs, treated or not with Cetuximab, were used on Cetuximab-treated CRC cells to verify the real influence of Cetuximab on the overall CAF protection. Indeed, Cetuximab treatment increased the basal protective activity of CAF supernatants, indicating an active effect of the drug on CAF secretome. The authors performed a cytokine-array analysis to identify the possible soluble mediators of this effect. Surprisingly, despite the different secretomes shown by three independent CAF primary cultures, the only increased cytokine was always EGF. These data were confirmed in ELISA and showed that two CRC cell lines and one normal colon-mucosa-derived fibroblasts did not upregulate EGF in response to Cetuximab in vitro. Curiously, the EGFR kinase inhibitor Erlotinib did not induce EGF in CAFs, suggesting that EGF release was not a direct response to EGFR pathway inactivity. EGFR triggering by Cetuximab could thus induce a specific CAF response. Could EGF alone be sufficient to reduce Cetuximab inhibitory effect on CRC cells? Tests of EGF on CRC epithelial cells and one 3D organoid culture, in the absence of CAF, showed a clear dose-dependent protection exerted by EGF against Cetuximab challenge, and the persistence of downstream Erk1-2 signaling. Thus, EGF can rescue CRC cells from Cetuximab inhibition. In line with these observations, Cetuximab-treated CAF-derived conditioned medium, neutralized by a specific anti-EGF antibody, was no longer able to increase the basic protection exerted by untreated CAF supernatants on CRC cells. This study suggests that CAFs, and possibly other fibroblast populations far from the tumor mass, could be the main mediators of a systemic resistance to Cetuximab by EGF upregulation.

The immunomodulatory activity of CAFs could also act as a supplementary mechanism of Cetuximab resistance. Cetuximab can activate macrophages and natural killer cells, triggering the antibody-dependent cellular cytotoxicity of tumor cells [10]. It has been recently shown, in HER2^+^ breast cancers, that a specific subset of CAFs can determine resistance to the anti-HER2 antibody Trastuzumab, lowering IL2 activity and causing immune exclusion [11]. A similar mechanism can be hypothesized for Cetuximab and CRC CAFs. These findings suggest that adoptive cell and anti-EGFR antibody immunotherapies should be planned to efficiently co-target CAF and CRC cells.

## 3. CAF in Radio-Chemoresistance

Radiotherapy (RT), while used to attack the tumor, also inevitably damages its stroma [12]. A typical effect of RT is the induction of CAF senescence. Senescent CAFs are characterized by a pro-fibrotic phenotype and the release of cytokines (i.e., TGFβ, CXCL12 and IGF1). TGFβ, in particular, can cause a self-amplification of signals, recruiting new fibroblasts from the periphery into the tumor stroma, which will be converted to CAFs. Thus, as a final effect, the CAF population supporting carcinoma cells will be increased, inducing epithelial–mesenchymal transition and cancer invasion. CAFs also contribute to chemotherapy resistance in different gastrointestinal tumors [13]. CAF subpopulations characterized by the expression of single different markers (αSMA, CXCL1, CXCL12, TGFβ and PAI1) have been linked to the poor outcome of gastric, esophageal and colorectal cancers under different chemotherapeutic regimens. The main mechanisms mediating CAF-driven chemoresistance are cytokine/growth factor production and non-coding RNAs transfer by exosomes. A representative example of a pleiotropic cytokine is CAF-derived IL6, which exerts several protective effects on tumor cells, activating the STAT3 (stem cell niche-trophic) and NFkB (proinflammatory) pathways. Coherently, the anti-IL6 receptor tocilizumab was able to abrogate CAF shielding in different experimental models [14]. An example of exosome-transferred resistance is CAF-derived miRNA miR-92a-3p, which can induce drug resistance in CRC cells, also sustaining invasion and stemness [15].

## 4. Conclusions

CAFs are relevant mediators of tumor resistance to radio-chemotherapy. Accordingly, some early attempts at targeting them are being tested in clinical trials.

In KRAS wild-type mCRC, under Cetuximab treatment, CAFs can be the most relevant source of increased EGF levels, reducing drug effectiveness. Cetuximab shows some activity against CRC metastases, where the tumor microenvironment has a different cellular composition, while the primary tumor is refractory to this treatment, helped by the powerful shielding of resident CAFs. While CAFs could be the principal therapeutic target to subvert Cetuximab resistance in mCRC with a wild-type EGFR pathway, the observation that the systemic EGF concentration can also be increased suggests a possible involvement of other fibroblast populations not confined to the tumor. This second mechanism could be particularly relevant when the primary tumor has been completely eradicated but CRC cells still persist in distant metastases.
